# Birth Weight Independently Affects Morbidity and Mortality of Extremely Preterm Neonates

**DOI:** 10.14740/jocmr2075w

**Published:** 2015-05-08

**Authors:** Apostolos Mamopoulos, Stamatios Petousis, John Tsimpanakos, Sophia Masouridou, Kelly Kountourelli, Chrysoula Margioula-Siarkou, Maria Papouli, David Rousso

**Affiliations:** a 3rd Academic Department Obstetrics & Gynaecology, Aristotle University of Thessaloniki, Medical School, “Hippokration” General Hospital, Thessaloniki, Greece; b Neonatal Intensive Care Unit, “Hippokration” General Hospital, Thessaloniki, Greece

**Keywords:** Extremely preterm, Neonatal morbidity, Neonatal mortality, Birth weight, Gestational age

## Abstract

**Background:**

Neonates born between 24 + 0 and 27 + 6 gestational weeks, widely known as extremely preterm neonates, present a category characterized by increased neonatal mortality and morbidity. Main objective of the present study is to analyze the effect of various epidemiological and pregnancy-related parameters on unfavorable neonatal mortality and morbidity outcomes.

**Methods:**

A retrospective study was performed enrolling cases delivered during 2003 - 2008 in our department. Cases of neonatal death as well as pathological Apgar score (≤ 4 in the first and ≤ 7 in the fifth minute of life), need for emergency resuscitation, respiratory disease syndrome (RDS), neonatal asphyxia, intraventricular hemorrhage (IVH) and neonatal death were recorded for neonates of our analysis. A multivariate regression model was used to correlate these outcomes with gestational week at delivery, maternal age, parity, kind of gestation (singleton or multiple), intrauterine growth restriction (IUGR), birth weight (BW), preterm premature rupture of membranes (PPROM), mode of delivery (vaginal delivery or cesarean section) and antenatal use of corticosteroids.

**Results:**

Out of 5,070 pregnancies delivered, 57 extremely preterm neonates were born (1.1%). Mean BW was 780.35 ± 176.0, RDS was observed in 93.0% (n = 53), resuscitation was needed in 54.4% (n = 31) while overall mortality rate was 52.6% (n = 30). BW was independently associated with neonatal death (P = 0.004), pathological Apgar score in the first (P = 0.05) and fifth minute of life (P = 0.04) as well as neonatal sepsis (P = 0.05).

**Conclusion:**

BW at delivery is independently affecting neonatal mortality and morbidity parameters in extremely preterm neonates.

## Introduction

Extremely preterm neonates (gestational age (GA) at birth below 27 + 6 weeks) represent a high-risk category characterized by increased rates of morbidity and mortality [1]. European studies report mortality rates ranging over 40%, while the proportion of neonates being discharged without serious morbidity from neonatal intensive care unit is reported to be only between 37% and 54% [2-4]. Furthermore, several studies have shown increased rates of long-term mental and psychomotor deficiencies of infants born at an extremely preterm gestational week, underlying that the study of mortality as well as of short and long-term morbidity aspects is an issue of great clinical significance [5-7].

Parental counselling regarding the optimal approach to such cases of extremely preterm labor is usually dependent on mortality and morbidity rates of each institution [8-10]. Several epidemiological and pregnancy-related parameters have been considered to potentially influence morbidity and mortality of extremely preterm neonates [8, 11, 12]. Predominantly, significant attention has been given to the gestational week at delivery time, all the therapeutic strategies being targeted to postponing a threatened labor to advanced gestational week. However, no definitive conclusions have been reached regarding the exact impact of gestational week itself or birth weight (BW), which is of course in its turn significantly affected by GA, on each neonatal morbidity outcome and especially on neonatal death, therefore implying the need to perform further studies on this critical clinical issue. Therefore, the main objective of the present study is to determine the impact of epidemiological and pregnancy-related parameters on the various short-term morbidity outcomes and, especially, on neonatal mortality.

## Methods

We performed a retrospective analysis of all the extremely preterm neonates (24 + 0-27 + 6 gestational weeks) that were delivered in our department during the years 2003 - 2008. Our department acts as a tertiary care obstetrical unit covering a major part of Northern Greece and working closely with a level 5 neonatal intensive care unit (NICU). Dating of the pregnancies was based on last menstrual period and on first trimester ultrasound measurements that were done while examining the nuchal translucency. In our study, we exclusively included extremely preterm neonates that were born between 24 + 0 and 27 + 6 gestational weeks for which precise evaluation of gestational week and close follow-up of pregnancy had been performed at least from the 20th gestational week. Institutional Review Board and University’s Ethical Committee approved the present study.

The epidemiological parameters examined were maternal age and parity whereas, obstetrical parameters included number of foetuses per pregnancy (singleton or multiple), kind of conception (natural or *in vitro* fertilization (IVF)) as well as antenatal complications including preterm premature rupture of membranes (PPROM), intrauterine growth restriction (IUGR) and fetal distress. Antenatal administration of corticosteroids, mode of delivery (vaginal delivery or cesarean section), neonatal BW and sex were also recorded for pregnancies enrolled in our analysis.

All the former parameters were correlated in a univariate regression model with neonatal morbidity outcomes and then, all the parameters that were statistically significant, were enrolled in a multivariate regression model. More specifically, morbidity parameters that were examined were pathological Apgar score in the first and in the fifth minute of life (≤ 4 and ≤ 7 respectively), need for emergency resuscitation at labor ward, neonatal asphyxia, respiratory distress syndrome (RDS), intraventricular hemorrhage (IVH), necrotizing enterocolitis (NEC) and neonatal sepsis. Neonatal mortality (death in the first 28 days of life) was also correlated in a univariate and then multivariate regression model with all the aforementioned pregnancy-related parameters as well as with RDS and IVH.

All data included in our analysis were extracted from the computerized medical databases used by both our department and the NICU. Both our department and the two NICUs record thoroughly elements of cases and use a double-checking system by two different physicians that have the responsibility to supervise the validity of records on a daily basis.

### Statistical analysis

Independent samples *t*-test was used for continuous and Chi-square for categorical data. Univariate and multivariate logistic backward regression models were used in order to correlate the latency period with the aforementioned parameters. Statistical analysis was performed using the Social Package for Statistical Science 17.0. Significance level was defined at P < 0.05.

## Results

Out of 5,070 pregnancies delivered in our department during the period 2003 - 2008, there were 57 extremely preterm live born neonates corresponding to 45 pregnancies (1.1%). Mean maternal age was 31.1 ± 5.8 and mean parity was 1.6 ± 0.8. Mean gestational week at delivery time was 25.8 ± 1.0 and mean BW was 780.4 ± 176.6. The majority of neonates were male (57.9%, n = 33) and the percentage of twins and triplets were 28.1% (n = 16) and 10.5% (n = 6) respectively. There were 13 neonates conceived by IVF (22.8%). Percentage of neonates with IUGR was 10.5% (n = 6) while PPROM was recorded in 34.5% of the cases (n = 19). Corticosteroids were administered in 33 neonates (57.9%), while cesarean section was the mode of delivery for 39 neonates (68.4%). Epidemiological and pregnancy-related characteristics are presented in Table 1.

**Table 1:** Demographics and Pregnancy-Related Characteristics in Extremely Preterm Neonates

Epidemiologic characteristics	Neonates (N = 57)
Maternal age (mean ± SD)	31.1 ± 5.8
Gestational week at delivery (mean ± SD)	25.8 ± 1.0
Parity (mean ± SD)	1.6 ± 0.8
Birth weight (mean ± SD)	780.4 ± 176.6
Conception	
Natural, n (%)	44 (77.2)
IVF, n (%)	13 (22.8)
Kind of pregnancy	
Singleton, n (%)	35 (61.4)
Twin, n (%)	16 (28.1)
Triplets, n (%)	6 (10.5)
Obstetrical complications	
IUGR, n (%)	6 (10.5)
PPROM, n (%)	19 (34.5)
Antenatal corticosteroids, n (%)	33 (57.9)
Mode of delivery	
Caesarean section, n (%)	39 (68.4)
Neonatal sex	
Male, n (%)	33 (57.9)
Female, n (%)	24 (42.1)

Neonatal mortality was 52.6% (n = 30). Regarding adverse neonatal outcomes, low Apgar scores during the first and fifth minute of life were observed in 47.4% (n = 27) and 49.1% (n = 28) respectively, while resuscitation in labor ward was demanded in 54.4% of cases (n = 31). Neonatal asphyxia rate was 14.0% (n = 31), RDS was diagnosed in 93.0% of cases (n = 53) during their stay in NICU while IVH was diagnosed in 21.1% (n = 12) of extremely preterm neonates. Neonatal mortality and morbidity rates for neonates of our study are shown in Table 2.

**Table 2:** Neonatal Mortality and Morbidity Outcomes of Neonates

Outcomes	Neonates (N = 57)
Apgar score in the first minute (mean ± SD)	4.1 ± 2.6
Apgar score in the fifth minute (mean ± SD)	6.6 ± 2.2
Apgar score ≤ 4 in the first minute, n (%)	27 (47.4)
Apgar score ≤ 7 in the fifth minute, n (%)	28 (49.1)
Respiratory distress syndrome, n (%)	53 (93.0)
Need for resuscitation, n (%)	31 (54.4)
Neonatal asphyxia, n (%)	8 (14.0)
Neonatal sepsis, n (%)	14 (24.6)
Necrotic enterocolitis, n (%)	3 (5.3)
Intraventricular hemorrhage, n (%)	12 (21.1)
Neonatal death, n (%)	30 (52.6)

Multivariate regression model indicated that both pathological Apgar scores in the first and fifth minute of life were independently associated with BW (P = 0.01 and P = 0.05 respectively). BW was also independently correlated with need for neonatal sepsis (P = 0.05). Male neonates had a 2.7-fold increased risk for RDS (P = 0.015). Finally, vaginal delivery was independently associated with need for resuscitation and neonatal asphyxia (P = 0.005). Results of univariate and multivariate regression models between pregnancy-related and neonatal morbidity parameters are presented in Tables 3 and 4.

**Table 3:** Univariate Regression Model Between Pregnancy-Related Parameters and Neonatal Morbidity Outcomes

	Low Apgar in first	Low Apgar in fifth	RDS	IVH	Resuscitation	Asphyxia	NEK	Sepsis
Gestational age	0.6 (0.3 - 0.99)*	0.6 (0.3 - 0.9)*	1.1 (0.4 - 2.8)	1.4 (0.7 - 2.7)	0.6 (0.3 - 0.99)*	0.7 (0.4 - 1.5)	1.8 (0.5 - 7.3)	1.3 (0.7 - 2.4)
Maternal age	1.0 (0.9 - 1.1)	1.0 (0.9 - 1.1)	1.0 (0.8 - 1.2)	1.0 (0.9 - 1.1)	1.0 (0.9 - 1.1)	1.0 (0.9 - 1.1)	1.2 (0.9 - 1.4)	1.0 (0.9 - 1.2)
Parity	0.8 (0.4 - 1.6)	0.9 (0.5 - 1.9)	0.8 (0.3 - 2.4)	0.4 (0.1 - 1.3)	1.1 (0.6 - 2.2)	1.6 (0.7 - 3.6)	0.6 (0.1 - 4.3)	1.1 (0.5 - 2.4)
IUGR	2.1 (0.4 - 12.3)	1.0 (0.2 - 6.8)	0.3 (0.03 - 3.6)	2.3 (0.9 - 2.8)	0.8 (0.2 - 4.5)	2.5 (0.8 - 3.0)	1.8 (0.7 - 2.2)	0.9 (0.8 - 1.1)
Multiple gestation	0.4 (0.1 - 1.1)	0.4 (0.1 - 1.1)	0.9 (0.6 - 1.4)	1.2 (0.3 - 4.3)	0.3 (0.09 - 0.90)*	0.9 (0.2 - 4.4)	3.4 (0.3 - 39.9)	0.9 (0.4 - 2.4)
PPROM	0.4 (0.1 - 1.1)	0.7 (0.2 - 2.3)	0.5 (0.07 - 3.9)	1.5 (0.4 - 5.5)	0.3 (0.10 - 1.1)	0.6 (0.1 - 3.2)	1.5 (0.1 - 17.4)	1.6 (0.5 - 5.6)
Corticosteroids	2.0 (0.7 - 6.1)	1.7 (0.5 - 5.6)	0.4 (0.04 - 4.5)	1.6 (0.4 - 6.1)	0.8 (0.3 - 2.2)	0.7 (0.2 - 3.1)	0.9 (0.1 - 10.8)	1.0 (0.3 - 3.3)
Mode of delivery	0.9 (0.3 - 2.8)	0.8 (0.2 - 2.9)	0.7 (0.07 - 7.3)	1.5 (0.4 - 6.4)	0.2 (0.1 - 0.8)*	0.2 (0.1 - 0.9)*	1.5 (0.4 - 6.4)	1.2 (0.3 - 4.5)
Birth weight	0.9 (0.9 - 0.98)*	0.9 (0.9 - 0.97)*	0.99 (0.98 - 1.01)	1.0 (0.997 - 1.004)	0.9 (0.9 - 1.01)	0.999 (0.995 - 1.003)	1.007 (0.999 - 1.014)	1.01 (1.01 - 1.04)*
Neonatal sex	0.80 (0.3 - 2.4)	1.7 (0.5 - 5.6)	2.7 (1.9 - 3.8)*	2.3 (0.6 - 8.4)	1.3 (0.5 - 3.8)	2.6 (0.6 - 12.3)	0.7 (0.1 - 7.9)	0. 5(0.1 - 1.7)

Values are expressed in odds ratio with 95% confidence intervals. *Parameters indicate statistical significance. IUGR: intrauterine growth restriction; PPROM: preterm premature rupture of membranes; RDS: respiratory distress syndrome; IVH: intraventricular hemorrhage; NEK: necrotic enterocolitis.

**Table 4:** Multivariate Regression Model Between Pregnancy-Related Parameters and Neonatal Morbidity Outcomes

	Low Apgar in first	Low Apgar in fifth	RDS	IVH	Resuscitation	Asphyxia	NEK	Sepsis
Gestational age	0.7 (0.4 - 1.3)	0.6 (0.3 - 1.3)	-	-	0.8 (0.4 - 1.5)	-	-	-
Maternal age	-	-	-	-	-	-	-	-
Parity	-	-	-	-	-	-	-	-
IUGR	-	-	-	-		-	-	-
Multiple gestation	-	-	-	-	0.4 (0.1 - 1.3)	-	-	-
PPROM	-	-	-	-	-	-	-	-
Corticosteroids	-	-	-	-	-	-	-	-
Mode of delivery	-	-	-	-	0.2 (0.05 - 0.9)*	0.2 (0.1 - 0.9)*	-	-
Birth weight	0.9 (0.9 - 0.98)*	0.9 (0.9 - 0.97)*	-	-	-	-	-	1.01 (1.01 - 1.04)*
Neonatal sex	-	-	2.7 (1.9 - 3.8)*	-	-	-	-	-

Values are expressed in odds ratio with 95% confidence intervals. *Parameters indicate statistical significance. IUGR: intrauterine growth restriction; PPROM: preterm premature rupture of membranes; RDS: respiratory distress syndrome; IVH: intraventricular hemorrhage; NEK: necrotic enterocolitis.

Regarding neonatal mortality, univariate logistic regression model demonstrated a significant association with BW, gestational week and IVH. However, multivariate model indicated that neonatal death was independently associated only with BW (P = 0.02) and IVH (P = 0.004). Results of univariate and multivariate regression models between pregnancy-related parameters and neonatal mortality are presented in Table 5.

**Table 5:** Univariate and Multivariate Regression Models Between Pregnancy-Related Parameters and Neonatal Mortality

	Univariate model mortality		Multivariate model mortality	
	P value	OR (95% CI)	P value	OR (95% CI)
Gestational age	0.02*	0.5 (0.3 - 0.9)*	0.70	0.6 (0.4 - 1.2)
Maternal age	0.47	1.0 (0.9 - 1.1)	-	-
Parity	0.16	0.6 (0.3 - 1.2)	-	-
IUGR	0.47	1.9 (0.3 - 11.4)	-	-
Multiple gestation	0.39	0.6 (0.2 - 1.8)	-	-
PPROM	0.44	0.6 (0.2 - 2.0)	-	-
Corticosteroids	0.73	1.2 (0.4 - 3.4)	-	-
Mode of delivery	0.78	1.2 (0.4 - 3.6)	-	-
Birth weight	0.007*	0.995 (0.991 - 0.999)*	0.007*	0.97 (0.90 - 0.99)*
Neonatal sex	0.73	0.8 (0.3 - 2.4)	-	-
RDS	0.91	1.1 (0.2 - 8.6)	-	-
IVH	0.03*	6.3 (1.2 - 31.8)*	0.02*	7.9 (1.4 - 45.1)*

Values are expressed in odds ratio with 95% confidence intervals. *Parameters indicate statistical significance. IUGR: intrauterine growth restriction; PPROM: preterm premature rupture of membranes; RDS: respiratory distress syndrome; IVH: intraventricular hemorrhage; NEK: necrotic enterocolitis.

## Discussion

Our study indicated that BW is the predominant factor independently affecting neonatal mortality as well as risk for low Apgar score and need for neonatal sepsis during NICU staying. Furthermore, it also indicated the potential impact mode of delivery may have on certain outcomes.

Neonatal mortality in our study was estimated to be 52.6%. A review of the published literature demonstrated a significant variability of extremely preterm neonates’ mortality. De Waal et al [1], in a large prospective population-based cohort study including 345 infants, reported comparable mortality of 48% whereas, relative rate was only 33% in a large longitudinal multicenter cohort study from Switzerland including 1,266 neonates [13]. The age definition criteria for extremely preterm neonates, the small number of such cases and the variations in the medical infrastructure might be the cause for such a variability. However, the majority of studies conclude that neonatal mortality before the 25th gestational week is significantly increased, predominantly ranging over 90% and this finding agrees with our results where mortality was almost 100% for neonates born at 24 weeks.

Even though BW is usually closely correlated to GA at birth, it is not clear which one of the two parameters affects mostly neonatal mortality. Despite the fact that our univariate model indicated that both gestational week and BW are correlated with neonatal mortality, when all the parameters were introduced in a multivariate regression model, it was only BW that was observed to be independently associated with mortality. The findings of the study by de Waal et al [1] agree with our results as, in their multivariate regression model, only BW and not gestational week was found to be independently associated with mortality. Similarly, Taeusch et al [14] indicate that survival rates were significantly associated with 50th percentile BW for each gestational week, reporting an increase from 26% for 522 g to 83% for 967 g. Even studies standing in favor of the significant impact of GA on mortality claim that this should not be the sole parameter on which our clinical decisions should be based, without considering the equally major impact of BW regarding neonatal mortality [15].

IVH may be another major determinant of neonatal mortality. Although not all relative studies enroll IVH in their multivariate regression model, there are studies standing in favor of the decisive role this parameter may have [15]. However, even when including IVH, the role of BW remains independently associated with the risk for neonatal death after NICU admission, upraising once again the demand to further elucidate on the role of BW on the final outcome.

Regarding neonatal morbidity outcomes, BW significantly affected the risk for low Apgar score and sepsis during NICU stay. Besides, vaginal delivery appears to affect the need for resuscitation and the risk for neonatal asphyxia. The role of BW has been thoroughly analyzed and seems to remain equally important for neonatal morbidity parameters as well [13]. The impact of mode of delivery, however, is another issue of controversy [16, 17]. There are studies standing in favor of our results. Schlapbach et al [12], when performing univariate regression model, have demonstrated a favorable impact of cesarean section on severe neonatal morbidity. Besides, Grant and Glazener, in the 2001 Cochrane Review [18] when comparing elective cesarean and expectant management of small neonates, have observed that babies in the elective group were less likely to suffer from RDS (odds ratio (OR): 0.43, 95% confidence interval (CI): 0.18 - 1.06), or to die but without this difference reaching statistical significance. They concluded, however, that there are no sufficient data to support elective cesarean as the absolute mode of delivery policy for small neonates. Despite the fact that the aforementioned meta-analysis does not examine extremely preterm neonates only, it seems that our observation that cesarean section may be beneficial in terms of neonatal asphyxia and need for resuscitation is rather supported by the already published literature. However, the yet unresolved issue of the optimal mode of delivery on this sensitive group of neonates is once again upraised. Besides, this is an issue in which no definitive conclusions may be easily stated because of the profound difficulties in organizing prospective randomized clinical trials.

Our study is not devoid of limitations. The retrospective character of the study as well as the relatively small sample size potentially consist the main ones. Furthermore, our study reports outcomes of neonates born between 2003 and 2008 and not the most recent data. However, it is one of the few studies enrolling extremely preterm neonates with a detailed follow-up including several neonatal morbidity parameters, in an effort to examine the impact of every epidemiological and pregnancy-related aspect to each adverse neonatal outcome. Moreover, it indicates the possible superiority of BW over GA as prognostic factor for neonatal morbidity and mortality, a conclusion which has not been clearly stated by other relative studies. Finally, we chose to study first the outcomes of neonates of an older period in order to compare this 5-year period with the modern one (2008 - 2013) after having completed the appropriate editing and statistical examination of modern period’s results.

In conclusion, our study demonstrated that BW might be an independent prognostic factor for mortality and morbidity of extremely preterm neonates, affecting significantly the decision of optimal management. Large prospective cohort studies and meta-analyses need to be performed in order to elucidate the exact impact of all epidemiological and obstetrical parameters on mortality and morbidity of extremely preterm neonates where the enigma of optimal management still remains unresolved. In any case, it is undoubtedly demanding to define the exact impact of each parameter, especially as the new era of prematurity caused by the increasing IVF performance poses a challenging clinical reality.
